# Influence of Different Feed Particle Sizes on the Growth Performance and Nutrition Composition in Crayfish, *Procambarus clarkii* Larvae

**DOI:** 10.3390/ani14152228

**Published:** 2024-07-31

**Authors:** Qingqing Jiang, Silei Xia, Zhiqiang Xu, Zhigang Yang, Lu Zhang, Guoxing Liu, Yu Xu, Aqin Chen, Xiaoru Chen, Fei Liu, Wenping Yang, Yebing Yu, Hongyan Tian, Yanmin Wu, Wuxiao Zhang, Aimin Wang

**Affiliations:** 1 College of Marine and Biology Engineering, Yancheng Institute of Technology, Yancheng 224051, China; jiangqq0812@163.com (Q.J.); susanxia1990323@126.com (S.X.); liufei@ycit.edu.en (F.L.); yangwenping@ycit.edu.cn (W.Y.); yuyebing2005@126.com (Y.Y.); tianhyy@outlook.com (H.T.); 18862008173@163.com (Y.W.); 2 Key Laboratory of Freshwater Aquatic Genetic Resources, Ministry of Agriculture and Rural Affairs, Shanghai Ocean University, Shanghai 201306, China; zgyang@shou.edu.cn (Z.Y.); aqchen@shou.edu.cn (A.C.); 3 Key Laboratory of Genetic Breeding and Cultivation for Freshwater Crustacean, Ministry of Agriculture and Rural Affairs, Freshwater Fisheries Research Institute of Jiangsu Province, Nanjing 210017, China; zhiqiangx@163.com (Z.X.); guoxingyzu@163.com (G.L.); jsgyxuyu@126.com (Y.X.); 4 Key Laboratory of Aquatic Nutrition and Smart Farming, Ministry of Agriculture and Rural Affairs, Healthy Aquaculture Key Laboratory of Sichuan Province, Tongwei Agricultural Development Co., Ltd., Chengdu 610093, China; zhangl21@tongwei.com (L.Z.); CHENXR@tongwei.com (X.C.)

**Keywords:** particle size, larval stage feed, *Procambarus clarkii*, growth performance, whole body composition, amino acids, fatty acids

## Abstract

**Simple Summary:**

This study investigated the relationship between feed particle size and growth performance or nutritional composition of crayfish (*Procambarus clarkii*) larvae. Our results showed that appropriate feed particle size could promote the growth and improve the nutritional value of crayfish. The critical values of suitable particle sizes for SGR and FCR were achieved at 0.55 mm and 0.537 mm. When the particle size of the feed exceeded 0.55 mm and 0.588 mm, the growth of crayfish reached the highest value and the FCR was the lowest. Where the feed particle size exceeds 0.55 mm and 0.588 mm, the growth and feeding of crayfish are unaffected. Compared to other groups, group C has the highest nutritional value. Therefore, we recommend that the appropriate food size for juvenile crayfish is 0.71–0.85 mm (group C).

**Abstract:**

A suitable feed size has a positive effect on animal feeding. For aquatic larvae, the correct feed size is very important for their growth. This experiment analyzed and compared the effect of different particle sizes of feed for larval stages on the growth performance, whole body composition, and muscle amino acid and fatty acid composition of crayfish. Five larval crayfish diets of different particle sizes, namely < 0.40 mm (Group A, control group), 0.40–0.50 mm (Group B), 0.71–0.85 mm (Group C), 0.90–1.00 mm (Group D) and 1.5 mm (Group E), were fed to 2000 crayfish (initial weight 0.0786 ± 0.0031 g) for 100 d. The results showed that as the particle size increased, final weight, weight gain (WG, *p* = 0.001) and specific growth rate (SGR, *p* = 0.000) of the crayfish tended to increase and then leveled off, with the control group being the lowest. The feed conversion ratio (FCR, *p* = 0.000) showed a decreasing and then equalizing trend with increasing particle size, but there was no significant difference between the groups except the control group. Broken-line regression analysis showed that the critical values for the appropriate particle feed size for crayfish larvae were 0.55 mm and 0.537 mm using SGR and FCR as indicators. Groups B, C and D had the highest crude protein content and were significantly higher than the control group (*p* = 0.001). Group E had the highest umami amino acid (UAA) and was significantly higher than the control group (*p* = 0.026). The content of isoleucine (Ile, *p* = 0.038) and phenylalanine (Phe, *p* = 0.038) was highest in group C and significantly higher than in the control group. Through principal component analysis, groups C and D were shown to contain leucine (Leu), glutamic (Glu), methionine (Met), valine (Val), histidine (His), Phe, and Ile levels significantly induced. The content of linoleic acid (C18:2n6, *p* = 0.000), linolenic acid (C18:3n3, *p* = 0.000), saturated fatty acid (SFA, *p* = 0.000), monounsaturated fatty acid (MUFA, *p* = 0.001), polyunsaturated fatty acid (PUFA, *p* = 0.000) and n-6 PUFA (*p* = 0.000) in group C was the highest and significantly higher than the control group. Principal component analysis showed that group C significantly induced the levels of C18:2n6, C18:3n3, DHA, EPA, n-3 PUFA and n-6 PUFA in muscle. Therefore, our results suggest that appropriate feed particle size can improve the growth performance and nutrient composition of crayfish. Based on the broken-line regression analysis of SGR and FCR, the critical values of optimal particle size for crayfish are 0.55 mm and 0.537 mm, and when the particle size exceeds these critical values (not more than 1.5 mm commercial feed), growth performance and FCR of the crayfish are no longer changed. Nevertheless, group C has high protein and low lipid content, as well as better nutrition with amino acids and fatty acids. Overall, combined with growth performance and nutrient composition, it is recommended that the particle size of the diet at the larval stage for crayfish is between 0.71 and 0.85 mm.

## 1. Introduction

With the rapid growth in aquatic feed production, feed waste and water pollution caused by low feed efficiency and poor water stability have attracted more and more attention. The larval stage of aquatic animals is a key stage in their early life cycle, and food in the larval stage is a key factor in the cultivation of seedlings. The nutrition and palatability of feed are directly related to seedling survival and growth [1,2]. Currently, research on micro-particle feed mainly focuses on feed formulation and feeding systems to improve feed conversion rate, while feed processing is less studied. Extrusion technology is the main method employed in the production of aquaculture feed [3]. The feed processing technology mainly includes crushing, mixing, pre-treatment, granulating and drying. However, the particle size of the feed in the pelleting process has a direct influence on the digestion and absorption of nutrients by aquatic animals and thus on their growth performance and nutrient composition [2,3]. Therefore, producing larval feed with appropriate particle size is an important way to ensure larval growth and improve feed conversion.

If the particle size of the feed is too large, it will not only cause difficulty with consumption, partial or total reduction in feed intake, but also lead to difficult digestion and absorption of nutrients [4]. On the contrary, if the feed size is too small, the feed will be lost as it dissolves more easily in water, reducing feed conversion and increasing breeding costs [5]. Studies have shown that the growth rate of aquatic animals is closely related to the particle size of the feed [6]. For example, feed particle size influences the growth of carp (*Cyprinus carpio*) [4], *Sander lucioperca* [7], and African catfish (*Clarias gariepinus*) [8]. Gao et al. [9] took gibel carp (*Carassius auratus gibelio*) as a research object and conducted tests using five different particle sizes feeds (104, 115, 163, 199 and 260 μm). They subsequently found that the FCR of the 104 μm group was lowest and larger pulverized feed size reduces intestinal health of fish. Aguado et al. [10] found that the specific growth rate of gilthead seabream (*Sparus aurata*) fed with 2 mm particle size feed was significantly increased and feed waste was reduced. In addition, it is unclear whether different particle sizes also affect the nutrient composition of aquatic animals, which is very important in the early development stages of aquatic animals.

*Procambarus clarkii* is an economically important aquaculture species in China. According to China Crayfish Industry Development Report (2023) [11], the total production of crayfish has reached 2.8907 million tons. High production means high feed input. Previous studies on crayfish mainly focused on nutrient requirements for each growth stage, feed formulation development, and finding the optimal feed protein source [12,13,14]. A comprehensive study on the effects of feed particle size on the growth performance and nutritional composition of crayfish has not been reported. However, to improve the quality of aquaculture feed, feed processing and feed physical properties should also be considered as important factors and analyzed together with basic research on feed nutrients. The particle sizes of the feed might influence the growth performance and nutritional composition of the crayfish. In this work, a study was carried out on the growth performance, whole body composition, muscle amino acid and fatty acid composition of crayfish fed different particle sizes of feed in the larval stage.

## 2. Materials and Methods

### 2.1. Experimental Design and Feeding Trial

Crayfish larvae used in the experiment were hatched from egg-bearing crayfish in the Yangzhong Base of Jiangsu Freshwater Fisheries Research Center, and the culture experiment was carried out in the Yangzhong Base. Before starting the experiment, crayfish larvae were temporarily reared in the recirculating water system and fed with a commercial diet powder containing 33% crude protein and 4% crude lipid (Tongwei Agricultural Development Co., Ltd. Sichuan, China) for two weeks. After the end of temporary rearing, healthy and vibrant crayfish larvae with the same size were divided into five treatment groups, each treatment group was quadruplicated in aquaculture tanks (2 m × 2 m × 0.5 m), and 100 crayfish larvae were randomly allocated to each aquaculture tank (initial weight 0.0786 ± 0.0031 g). The commercial feed (proximate composition: moisture = 9.21 ± 0.03%, crude protein = 36.77 ± 0.15%, crude lipid = 4.97 ± 0.09%, ash 13.53 ± 0.18%) corresponding to the temporary feeding period and having an initial particle size of 1.5 mm was crushed and then sieved to the appropriate particle size. The five treatment groups were: <0.40 mm (group A, control group), 0.40–0.50 mm (group B), 0.71–0.85 mm (group C), 0.90–1.00 mm (group D) and 1.5 mm (group E).

During the 100-day feeding trial, all groups were fed at 5% of their average body weight every day (30% of total daily feed, 8:00 am; 70% of total daily feed, 4:00 pm), and the feeding rate was adjusted for body weight every two weeks. Because the crayfish are so small, uneaten feed must be collected very carefully with a siphon and mortality was recorded daily. The aquaculture system used in this experiment was an indoor recirculating water system. Dissolved oxygen content in the water was 5.0–6.0 mg/L, the water temperature was maintained at 15 ± 3 °C, the content of nitrite in the water was not more than 0.1 mg/L, the ammonia nitrogen concentration was not more than 0.02 mg/L, the pH was between 7.2 and 7.5. Photoperiod was the natural light–dark cycle throughout the experimental period (February–May).

### 2.2. Sample Collection

At the end of the trial, the crayfish were fasted for 24 h. Before sampling, each group was counted to determine survival and the total weight of each group was used to calculate weight gain and specific growth rates. After measuring the body weight and length of six crayfish in each tank, the muscles were dissected and weighed and used to calculate meat content. The tail muscles of crayfish were sampled and stored at −80 °C for the determination of amino acids and fatty acids. The remaining crayfish were sampled and sorted at −20 °C for whole-body composition analysis.

### 2.3. Index Determination Method

#### 2.3.1. Growth Performance

The calculation equations of the growth performance in this study are as follows:
Survival rate, SR = 100% × (K_t_/K_0_)Weight gain, WG = 100% × (W_t_ − W_0_)/W_0_Specific growth rate, SGR = 100% × (lnW_t_ − ln W_0_)/tFeed conversion ratio, FCR = 100% × G_d_/(W_t_ − W_0_)Condition factor, CF = 100 × W_t_/L^3^Feed intake (FI, g/g of crayfish/day) = feed consumption/((W_t_ + W_0_)/2 × the experimental duration in days)Meat content, MC = 100% × G_m_/W_t_
where, K_t_ is the final number of crayfish, K_0_ is the initial number of crayfish, W_t_ is the final weight, W_0_ is the initial weight, it is the experimental days, G_d_ is the food ration, L is the final body length, and G_m_ is the muscle weight.

#### 2.3.2. Determination of Whole-Body Composition

The conventional components of test feed and whole crayfish were determined according to the method described by You et al. [15]. Moisture was determined by the constant weight drying method at 80 °C, crude lipid content by Soxhlet extraction method, and crude protein (N × 6.25) using the Kjeldahl method. The sample was burned in a muffle furnace at 550 °C for 6 h and the crude ash content was determined by the carbonization method.

#### 2.3.3. Determination of Muscle Amino Acids and Fatty Acids

Amino acids were determined according to the method of Ma et al. [16] using a high-speed amino acid analyzer (LA8080, Hitachi, Ltd., Tokyo, Japan). A sample of approximately 0.2 g was weighed into a 50 mL hydrolysis tube, 20 mL of 1 + 1 HCL was added, and the sample was hydrolyzed in an electric blast drying oven at 110 °C for 22 h. The sample was removed, cooled, and then transferred to a 25 mL cuvette for volume determination. Exactly 100 μL of the sample was placed into a 15 mL centrifuge tube, put it in the vacuum drying oven, dry at 60 °C for 2 h (drying all the solvent), and after drying, mixed with distilled water to 0.5 mL and run through the 0.45 μm organic membrane on the machine.

Fatty acids were analyzed by gas chromatography (GC-2010, Shimadzu, Japan) according to the method of Chen et al. [17]. A sample of approximately 0.2 g was taken randomly and added to 2 mL of a petroleum ether-ether mixture (*v*/*v*, 1:1) and allowed to sit overnight. Then, 1.5 mL of a 2% KOH-methanol solution was added, mixed, and the sample was allowed to stand for 1 h. After centrifugation at 11,800× *g* for 10 min, the supernatant was removed and 1.5 mL of BF3-CH3OH at a mass fraction of 14% was added. The fatty acids were then methylated by placing them in a 55 °C water bath for 30 min. After cooling, added 1.5 mL each of hexane and saturated NaCl solution (extraction process) and left to stratify at 4 °C, removed the supernatant completely, filtered through an organic phase filter membrane with a pore size of 0.22 μm and then analyzed by gas chromatography.

### 2.4. Statistical Analysis

The experimental data were analyzed using SPSS 25.0 for biological statistics. The assumptions of normality and mean square deviation were confirmed before any statistical analysis. One-way analysis of variance (ANOVA) followed by Duncan’s multiple range test was used to determine whether different levels of substitution had a significant effect on the measurements. The level of significance was set as *p* < 0.05. Statistics were expressed as mean ± SEM (standard error of the mean) and differences were considered significant at *p* < 0.05. Principal component analysis and plotting using origin 2018.

## 3. Results

### 3.1. Growth Performance

**Figure 1:**
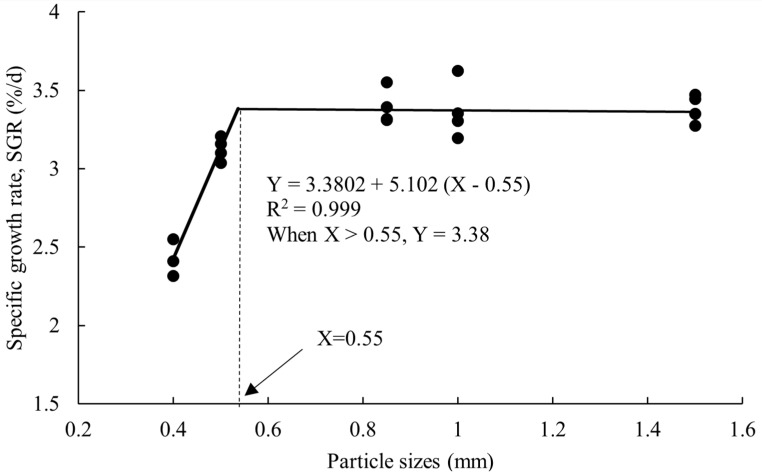
Broken–line regression analysis between dietary particle sizes of larval feed and SGR of *P. clarkii*.

**Figure 2:**
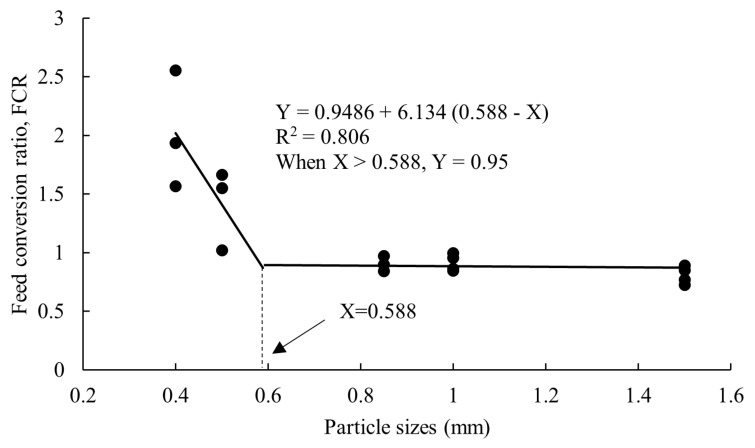
Broken–line regression analysis between dietary particle sizes of larval feed and FCR of *P. clarkii*.

The growth performance of crayfish after 100 days of feeding the five larval feeds are shown in Table 1. There were no significant differences in SR, CF and MC between the five groups (*p* > 0.05). WG, SGR, and the final mean weight of groups C, D and E were significantly higher than that of the control group (*p* < 0.05), FCR was significantly lower than the control group (*p* < 0.05), and there was no significant difference between groups B, C, D and E (*p* > 0.05). According to the broken-line regression analysis of SGR and FCR, at particle sizes of 0.55 mm and 0.588 mm, the SGR of crayfish was the highest (Figure 1) and the FCR of crayfish was the lowest (Figure 2).

### 3.2. Whole-Body Composition

The whole-body composition of crayfish after 100 days of feeding the five larval feed is shown in Table 2. There was no significant difference in moisture and crude ash content between the five groups (*p* > 0.05). Compared to the control group, the crude protein content in groups B, C and D was significantly increased (*p* < 0.05) and the crude lipid content was significantly reduced in groups B, C, D and E (*p* < 0.05).

### 3.3. Amino Acids Composition and Principal Component Analysis

**Figure 3:**
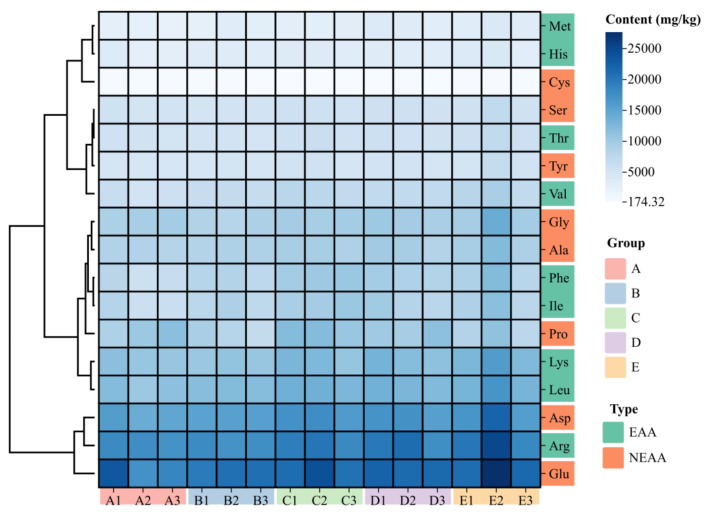
Heat map of the amino acid composition of *P. clarkii* muscle. Note: The darker the color in the grid, the higher the content.

The amino acid composition of the muscle of crayfish after 100 days of feeding the five experimental diets is shown in Table 3 and Figure 3. In total, 17 amino acids were detected in all 5 groups of crayfish, of which 9 were essential amino acids (EAA) and 8 were non-essential amino acids (NEAA). Glutamate (Glu) content was the highest, followed by Arg, but there was no significant difference between the five groups (*p* > 0.05, Table 3). The content of cysteine (Cys) in group C was significantly lower than that in control group and group E (*p* < 0.05). The content of isoleucine (Ile), leucine (Leu), methionine (Met), valine (Val), phenylalanine (Phe), lysine (Lys), aspartate (Asp), tyrosine (Tyr) and umami amino acids (UAA) in group E was significantly higher than that of the control group (*p* < 0.05), but was not significantly different from that of group C and group D (*p* > 0.05). There were no significant differences in the content of EAA, NEAA and other amino acids between the five groups (*p* > 0.05).

**Figure 4:**
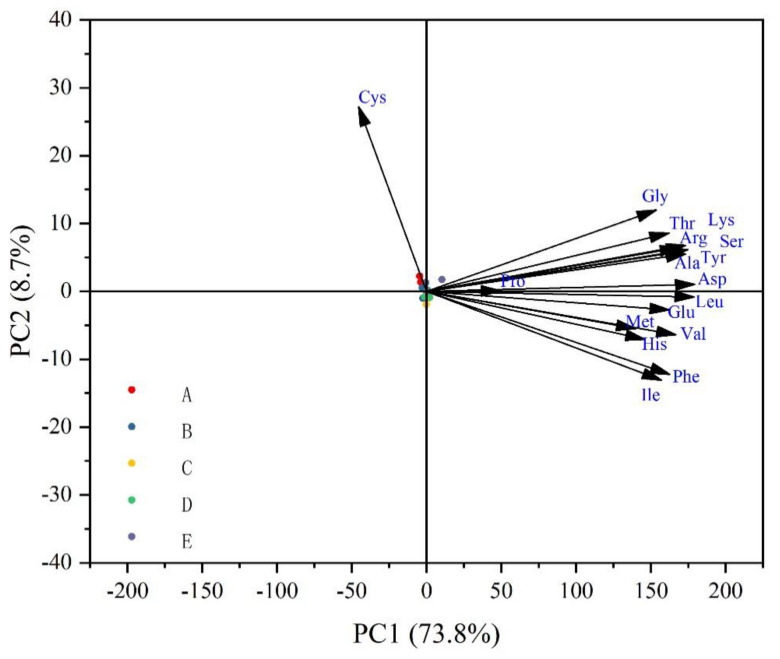
Principal component analysis (PCA) of all measured variables (amino acid composition) at five particle sizes of larval feed (●A, ●B, ●C, ●D, ●E). Arrows represent variables.

The principal component analysis (PCA) of the amino acids of muscle after feeding the five test diets to crayfish for 100 d is shown in Figure 4. The analysis showed that 73.8% of the total variance was attributable to PC1 and 8.7% to PC2, with the PC2 axis indicating the separation between group A and groups C and D. Among them, groups C and D exhibited significantly increased Phe and Ile levels in muscle.

### 3.4. Fatty Acids Composition and Principal Component Analysis

**Figure 5:**
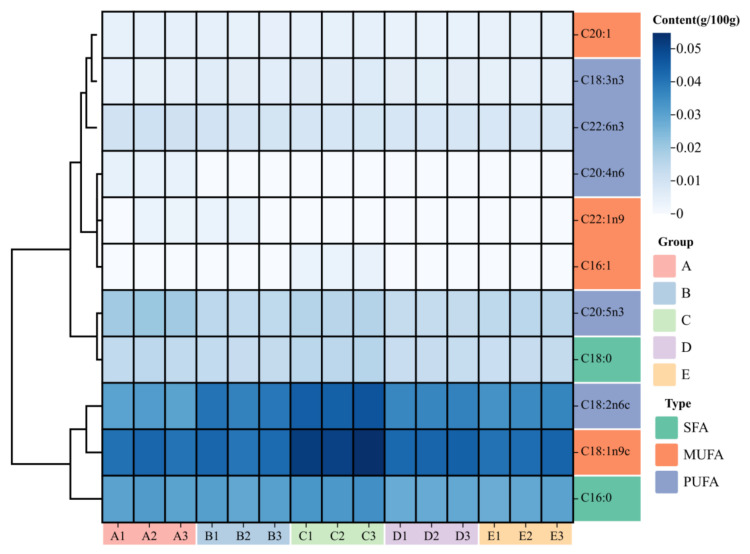
Heat map of the fatty acid composition of *P. clarkii* muscle. Note: The darker the color in the grid, the higher the content.

The fatty acid composition of the muscle of crayfish after 100 days of feeding the five experimental diets is shown in Figure 5. The concentration of C18:1n9 and C18:2n6 (linoleic acid, LA) in group C was the highest and significantly higher than all groups (*p* < 0.05, Table 4). As shown in Table 4, the concentration of C16:0 (palmitic acid, PA), C18:0 (stearic acid, SA), C18:3n3 (linolenic acid, LNA), SFA, MUFA and PUFA in group C were significantly higher than all groups (*p* < 0.05). The n-6 PUFA was highest in group C and was significantly different from the control group (*p* < 0.05); n-3 PUFA was highest in control group, followed by group C.

**Figure 6:**
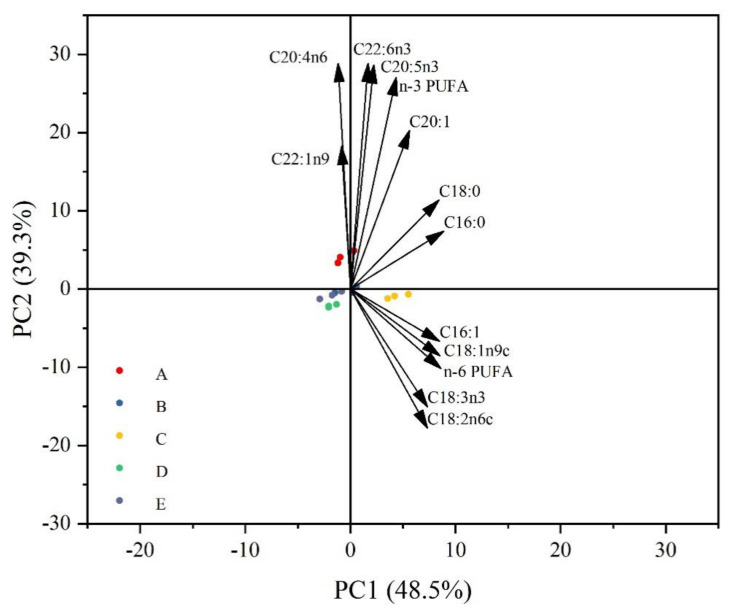
Principal component analysis (PCA) of all measured variables (fatty acid composition) at five particle sizes of larval feed (●A, ●B, ●C, ●D, ●E). Arrows represent variables.

The principal component analysis (PCA) of muscle fatty acids after feeding the five larval feed to crayfish for 100 d is shown in Figure 6. The analysis showed that 48.5% of the total variance was attributable to PC1 and 39.3% to PC2. The PC1 axis indicated the separation between groups A and C, and the PC2 axis indicated the separation between groups A and B, C, D, and E. The PC1 axis indicated the separation between groups A and C, and the PC2 axis indicated the separation between groups A and C. In group A, C20:4n6 and C22:1n9 levels were induced in muscle, while in group C, C18:2n6, C18:3n3, DHA, and EPA, n-3 PUFA and n-6 PUFA levels were induced in muscle.

## 4. Discussion

Some fish studies have found that the relationship between feed particle size and fish growth follows a conic model [18]. In this study, compared with the control group, WG, SGR, and final mean weight of groups C, D and E were higher and FCR was lower. According to the broken-line regression analysis of SGR and FCR, the critical values for the appropriate particle size of feed at the larval stage for crayfish were 0.55 mm and 0.588 mm. Mattila et al. [18] investigated the effects of six particle sizes on the growth performance of *Sander lucioperca* and found that the specific growth rate decreased with increasing feed particle size. Cho et al. [19] studied and compared the effects of different extruder pressures and particle sizes on the growth performance of rockfish (*Sebastes schlegeli*) and showed that low extruder pressure in the manufacturing process and small raw material particles were more effective in improving fish body growth than high pressure and large particles. When feeding, the particle size of the feed might affect the growth of fish through factors such as difficulty associated with catching and ingesting, the size of the surface area and the speed of gastrointestinal emptying. When the feed particle size is small, the feed surface area is large, the dissolution rate is high, and the nutrient composition is dissolved [8]. Smaller grains accelerated gastrointestinal emptying, resulting in shorter digestion and absorption time and reduced digestive efficiency. At the same time, fish must expend more energy to achieve the same intake as when fed with larger particle feed [2]. Since the feeding behavior of crayfish is to hold food, the particle size of the feed has a major influence on its intake. The speed of gastrointestinal emptying of gilthead sea bream (*Sparus aurata*) is also affected by a specific feeding behavior (chewing) like crayfish influenced by the particle size of the feed [20]. This is why differences in grain size can be observed when different grain sizes of feed enter the digestive tract of crayfish. In general, as fish and crabs grow, the feeding or digestive organs such as the mouth, stomach and intestines gradually become larger, and the optimal food particle size may also increase [5]. Because the culture time of crayfish was longer, the growth performance of crayfish was better when the particle size was larger. Unlike fish, crayfish rely on pincers to feed, and food particle size above a certain limit may not affect crayfish feeding. This explains why feeding above a certain threshold in this study did not affect the SGR and FCR of crayfish.

When feeding crayfish, apart from achieving better growth performance, nutrient composition represents another important indicator. Aquatic animals consume food, and digestion and absorption of nutrients are part of the metabolism for growth, the other part is deposited in the body, and the food affects the animal’s nutrient deposition and retention. There was no effect of different particle sizes on moisture and crude ash, like most nutrient studies, and none of them had a significant effect on muscle moisture [21,22,23]. The main nutritional component of crayfish is muscle, and the nutritional content in muscle is mainly composed of proteins and lipids. As an important material basis for life, protein is an important indicator of nutritional value. In this study, groups B, C and D contain the highest crude protein content. As the feed particle size increases, an initial increase followed by a decrease in crude protein composition is observed, indicating that different particle sizes have a significant influence on the crude protein composition of crayfish and that the appropriate particle size can promote the accumulation of proteins in the body. This is probably because the appropriate particle size was easier to digest and absorb [24]. This may also be related to the dissolving of particle sizes that are too small. In the present study, crude lipid content was found to be lowest in groups C, D and E and was negatively affected by particle size. This may be because when the feed is crushed into small particle sizes, the starch in the feed is saccharified and the sugar content in the feed increases. Subsequently, the excess sugar in the body is converted into lipids through glycolysis and other pathways and then deposited in the liver and muscles, which increases the body’s lipid content [25].

Amino acids, as basic structural and functional units of protein composition, are one of the key indicators for assessing the nutritional value of meat, which can directly affect the sense of taste and indirectly participate in the formation of flavor [26]. The taste of muscles depends on the composition and content of umami amino acids in the muscle [27]. EAA content is an important nutritional index for muscle production and also an important substance for catabolism in the larval stage [28]. Since some organs and structures of juvenile aquatic animals are still in the process of development, amino acids are more easily absorbed, thereby promoting the growth and development of larvae. It also promotes the secretion of pancreatic protease, which helps digest bait [29]. In this study, the content of Ile and Phe in group C was significantly higher than that of the control group, and groups C and D significantly increased Lue, Glu, Met, Val, histidine (His), Phe, and Ile in the muscles. Our results show that group C appears to have better amino acid nutritional value, which had a more suitable particle size. Possibly because smaller food particles accelerate gastrointestinal emptying, resulting in shorter digestion and absorption time and lower digestive efficiency [10]. Thus, the appropriate particle size influences the amino acid composition of crayfish during early development. In addition, as fish and shrimp grow, their mouth, stomach, intestines and other ingestion and digestive organs gradually become larger, which may also increase the optimal feed particle size [5].

There is widespread agreement about the importance of fatty acid composition to the public [30]. The fatty acid profile also influences the muscle quality and taste of fish, especially the unsaturated fatty acid content [31,32]. Among PUFAs, DHA and EPA are beneficial not only for the growth and reproduction of fish but also for human health [33,34]. Therefore, the composition and content of PUFA were crucial for estimating the nutritional value of fatty acids [35]. In addition, highly unsaturated fatty acid (HUFA) is an essential fatty acid for crustaceans and plays an irreplaceable role in the survival, growth and molting of early aquatic animals [36]. The composition of muscle fatty acids was related to the type of diet [37,38]. In this study, the C18:2n6, C18:3n3, MUFA and PUFA contents in group C were significantly higher than other groups, and it was found that group C increased the concentrations of C18:2n6, C18:3n3, DHA, EPA, n-3 PUFA, and n-6 PUFA in muscle as shown via PCA analysis. This indicates that proper particle size can improve fatty acid nutrition during the early development of crayfish and provide essential fatty acids for growth and development. Meanwhile, group C has higher nutritional value and is more beneficial to human health. This may be because the appropriate particle size is more favorable for digestion and absorption by crayfish.

## 5. Conclusions

Overall, feed particle size influences the growth performance and nutritional composition of crayfish larvae. The critical values of suitable particle sizes for SGR and FCR were achieved at 0.55 mm and 0.588 mm. The content of crude protein, some essential amino acids and fatty acids in groups C, D and E were greater than the control group. Nevertheless, group C exhibited high protein and low lipid content, as well as better nutrition with amino acids and fatty acids. Based on the above results, the following conclusions were made: (1) When the particle size of the feed did not exceed 0.55 mm and 0.588 mm, the growth of crayfish continued to increase as the feed size increased, but the feed coefficient continued to decrease. When the particle size of the feed reached or exceeded 0.55 mm and 0.588 mm, the growth of crayfish reached the highest value and the FCR was the lowest; (2) If the feed particle size exceeds 0.55 mm and 0.588 mm, the growth and feeding of crayfish are unaffected; (3) Compared to other groups, group C demonstrates the highest nutritional value. Therefore, we recommend that the appropriate food size for juvenile crayfish is 0.71–0.85 mm (group C).

## Figures and Tables

**Table 1:** Effects of five particle sizes of larval feed on growth performance of *P. clarkii*.

Item	A	B	C	D	E	*p* Value
W_t_ (g)	1.148 ± 0.255 ^a^	1.795 ± 0.065 ^b^	2.353 ± 0.136 ^b^	2.316 ± 0.222 ^b^	2.330 ± 0.104 ^b^	0.001
WG (%)	1355.471 ± 324.521 ^a^	2180.852 ± 83.579 ^ab^	2888.868 ± 173.579 ^b^	2843.702 ± 282.736 ^b^	2860.242 ± 132.481 ^b^	0.001
SGR (%/d)	2.615 ± 0.196 ^a^	3.125 ± 0.037 ^b^	3.393 ± 0.056 ^b^	3.369 ± 0.0913 ^b^	3.385 ± 0.045 ^b^	0.000
FCR	2.245 ± 0.304 ^b^	1.274 ± 0.196 ^a^	1.034 ± 0.132 ^a^	0.915 ± 0.036 ^a^	0.809 ± 0.038 ^a^	0.000
CF (g·cm^−3^)	2.382 ± 0.104	2.377 ± 0.060	2.370 ± 0.048	2.526 ± 0.0611	2.490 ± 0.062	0.358
MC (%)	15.249 ± 0.708	15.978 ± 0.651	16.200 ± 0.529	16.951 ± 0.655	15.738 ± 0.452	0.285
FI	1.983 ± 0.308 ^b^	1.176 ± 0.042 ^a^	0.911 ± 0.047 ^a^	0.938 ± 0.078 ^a^	0.917 ± 0.041 ^a^	0.000
SR (%)	55.00 ± 4.916	56.25 ± 8.045	51.50 ± 6.886	57.00 ± 5.745	62.00 ± 0.577	0.788

Notes: W_t_ = final body weight; WG = weight gain; SGR = specific growth ratio; FCR = Feed conversion ratio; CF = Condition factor; MC = Meat content; FI = feed intake. Values are presented as mean ± SEM (*n* = 4). Values with different superscripts in the same row are significantly (*p* < 0.05) different.

**Table 2:** Effects of five particle sizes of larval feed on whole body composition of *P. clarkii*.

Item (%)	A	B	C	D	E	*p* Value
Moisture	66.79 ± 1.18	65.01 ± 0.78	66.02 ± 1.86	63.21 ± 3.62	67.27 ± 2.82	0.741
Crude protein	24.20 ± 2.06 ^a^	30.77 ± 1.28 ^b^	29.53 ± 0.42 ^b^	32.89 ± 1.42 ^b^	22.58 ± 0.88 ^a^	0.001
Crude lipid	8.64 ± 0.24 ^c^	7.00 ± 0.41 ^b^	5.29 ± 0.44 ^a^	4.37 ± 0.11 ^a^	5.38 ± 0.27 ^a^	0.000
Crude ash	7.71 ± 1.34	6.04 ± 0.20	6.61 ± 0.45	7.64 ± 0.43	8.42 ± 0.48	0.202

Note: Values are presented as the mean ± SEM (*n* = 3). Values with different superscripts in the same row are significantly (*p* < 0.05) different.

**Table 3:** Effects of five particle sizes of larval feed on muscle amino acids of *P. clarkii*.

Item (mg/g)	A	B	C	D	E	*p* Value
Arg	17.74 ± 0.27	17.70 ± 0.22	19.37 ± 0.60	19.69 ± 1.04	21.27 ± 2.00	0.170
His	3.12 ± 0.32	3.47 ± 0.07	3.57 ± 0.11	3.44 ± 0.04	3.81 ± 0.31	0.291
Ile	7.01 ± 0.69 ^a^	8.19 ± 0.48 ^ab^	9.94 ± 0.32 ^b^	8.89 ± 0.62 ^ab^	10.40 ± 1.38 ^b^	0.038
Leu	11.43 ± 0.51 ^a^	12.10 ± 0.09 ^a^	13.40 ± 0.30 ^ab^	12.97 ± 0.32 ^a^	15.11 ± 1.88 ^b^	0.032
Met	2.48 ± 0.11 ^a^	3.02 ± 0.22 ^ab^	3.03 ± 0.04 ^ab^	3.54 ± 0.23 ^bc^	3.64 ± 0.22 ^c^	0.007
Val	5.92 ± 0.37 ^a^	6.82 ± 0.11 ^ab^	7.67 ± 0.27 ^bc^	7.24 ± 0.04 ^bc^	8.31 ± 0.64 ^c^	0.014
Phe	6.92 ± 0.67 ^a^	8.24 ± 0.27 ^ab^	10.25 ± 0.22 ^b^	9.11 ± 0.39 ^ab^	9.85 ± 1.25 ^b^	0.038
Lys	11.14 ± 0.31 ^a^	10.91 ± 0.17 ^a^	12.16 ± 0.62 ^ab^	12.33 ± 0.52 ^ab^	14.47 ± 1.61 ^b^	0.034
Thr	5.43 ± 0.23 ^a^	5.23 ± 0.08 ^a^	5.95 ± 0.20 ^ab^	5.71 ± 0.11 ^a^	6.56 ± 0.61 ^b^	0.042
Ala	8.67 ± 0.10	8.88 ± 0.13	9.42 ± 0.18	9.42 ± 0.44	10.34 ± 1.00	0.233
Gly	9.55 ± 0.34	8.75 ± 0.25	9.78 ± 0.10	9.90 ± 0.28	11.26 ± 1.45	0.276
Asp	15.00 ± 0.56 ^a^	15.54 ± 0.17 ^a^	17.15 ± 0.46 ^ab^	16.73 ± 0.54 ^ab^	18.36 ± 0.98 ^b^	0.018
Glu	19.80 ± 18.90	20.62 ± 0.39	22.11 ± 1.08	21.78 ± 0.25	23.58 ± 2.05	0.390
Tyr	4.81 ± 0.05 ^a^	4.89 ± 0.13 ^a^	5.27 ± 0.17 ^ab^	5.16 ± 0.14 ^ab^	6.12 ± 0.72 ^b^	0.044
Ser	5.40 ± 0.13	5.26 ± 0.05	5.69 ± 0.12	5.72 ± 0.11	6.12 ± 0.54	0.239
Pro	10.46 ± 0.80 ^ab^	8.27 ± 0.18 ^a^	11.68 ± 0.49 ^b^	10.52 ± 0.56 ^ab^	9.24 ± 1.08 ^ab^	0.047
Cys	0.39 ± 0.02 ^b^	0.36 ± 0.05 ^b^	0.19 ± 0.02 ^a^	0.25 ± 0.04 ^a^	0.38 ± 0.03 ^b^	0.029
^1^ EAA	75.13 ± 3.10	75.65 ± 1.12	85.33 ± 1.55	88.88 ± 8.41	91.10 ± 8.26	0.202
^2^ NEAA	71.91 ± 4.01	72.54 ± 0.17	81.26 ± 2.19	79.55 ± 1.07	85.06 ± 8.53	0.224
^3^ UAA	62.59 ± 4.83 ^a^	66.91 ± 0.66 ^ab^	73.98 ± 1.60 ^ab^	72.10 ± 1.83 ^ab^	79.19 ± 4.09 ^b^	0.026

Note: Arg, arginine; His, histidine; Ile, isoleucine; Leu, leucine; Met, methionine; Val, valine; Phe, phenylalanine; Lys, lysine; Thr, threonine; Ala, alanine; Gly, glycine; Asp, aspartic; Glu, glutamic; Tyr, tyrosine; Ser, serine; Pro, proline; Cys, cysteine; ^1^ EAA, essential amino acids (including Arg, His, Ile, Leu, Met, Val, Phe, Lys, Thr); ^2^ NEAA, non-essential amino acids (including Ala, Gly, Asp, Glu, Tyr, Ser, Pro, Cys). ^3^ UAA, umami amino acid (including Phe, Ala, Gly, Asp, Glu, Tyr). Values are presented as mean ± SEM (*n* = 3). Values with different superscripts in the same row are significantly (*p* < 0.05) different.

**Table 4:** Effects of five particle sizes of larval feed on muscle fatty acids of *P. clarkii*.

Item (g/100 g)	A	B	C	D	E	*p* Value
C16:0	0.031 ± 0.0006 ^b^	0.030 ± 0.0006 ^b^	0.034 ± 0.0006 ^c^	0.028 ± 0.0002 ^a^	0.029 ± 0.0007 ^ab^	0.001
C16:1	ND	ND	0.004 ± 0.0001	ND	ND	ND
C18:0	0.014 ± 0.0003 ^b^	0.013 ± 0.0003 ^a^	0.016 ± 0.0003 ^c^	0.013 ± 0.0001 ^a^	0.013 ± 0.0003 ^a^	0.000
C18:1n9	0.042 ± 0.0008 ^a^	0.042 ± 0.0010 ^a^	0.053 ± 0.0011 ^b^	0.044 ± 0.0004 ^a^	0.042 ± 0.0009 ^a^	0.000
C18:2n6 (LA)	0.031 ± 0.0005 ^a^	0.039 ± 0.0010 ^c^	0.046 ± 0.0008 ^d^	0.037 ± 0.0003 ^b^	0.036 ± 0.0009 ^b^	0.000
C18:3n3 (LNA)	0.005 ± 0.0001 ^a^	0.006 ± 0.0001 ^b^	0.007 ± 0.0002 ^c^	0.006 ± 0.0001 ^b^	0.005 ± 0.0001 ^a^	0.000
C20:1	0.005 ± 0.0001 ^c^	0.005 ± 0.0001 ^bc^	0.005 ± 0.0001 ^c^	0.004 ± 0.0001 ^a^	0.004 ± 0.0001 ^b^	0.001
C22:1n9	0.003 ± 0.0001	0.004 ± 0.0001	ND	ND	ND	ND
C20:4n6 (AA)	0.004 ± 0.0001	ND	ND	ND	ND	ND
C20:5n3 (EPA)	0.020 ± 0.0004 ^e^	0.015 ± 0.0003 ^b^	0.016 ± 0.0002 ^d^	0.013 ± 0.0001 ^a^	0.015 ± 0.0004 ^c^	0.000
C22:6n3 (DHA)	0.011 ± 0.0002 ^d^	0.010 ± 0.0002 ^c^	0.009 ± 0.0001 ^b^	0.009 ± 0.0001 ^a^	0.009 ± 0.0002 ^ab^	0.000
SFA	0.045 ± 0.0009 ^b^	0.044 ± 0.0009 ^ab^	0.049 ± 0.0009 ^c^	0.041 ± 0.0003 ^a^	0.042 ± 0.0010 ^a^	0.000
MUFA	0.049 ± 0.0018 ^a^	0.049 ± 0.0013 ^a^	0.062 ± 0.0023 ^b^	0.050 ± 0.0028 ^a^	0.047 ± 0.0001 ^a^	0.001
PUFA	0.071 ± 0.0011 ^b^	0.070 ± 0.0016 ^b^	0.078 ± 0.0013 ^c^	0.065 ± 0.0008 ^a^	0.065 ± 0.0015 ^a^	0.000
n-3 PUFA	0.036 ± 0.0011 ^d^	0.030 ± 0.0006 ^b^	0.033 ± 0.0005 ^c^	0.029 ± 0.0006 ^ab^	0.028 ± 0.0002 ^a^	0.000
n-6 PUFA	0.035 ± 0.0005 ^a^	0.039 ± 0.0010 ^b^	0.046 ± 0.0008 ^c^	0.037 ± 0.0003 ^a^	0.036 ± 0.0009 ^a^	0.000

Note: LA, linoleic acid; LNA, linolenic acid; AA, arachidonic acid; EPA, eicosapentaenoic acid; DHA, docosahexaenoic acid; SFA, saturated fatty acid; MUFA, monounsaturated fatty acid; PUFA, polyunsaturated fatty acid. Values are presented as mean ± SEM (*n* = 3). Values with different superscripts in the same row are significantly (*p* < 0.05) different.

## Data Availability

The data presented in this study are available on request from the corresponding author.
